# Preliminary Concurrent Validity of Abbreviated App-Based Frequency and Duration Pattern Tests in Normal-Hearing Adults

**DOI:** 10.3390/audiolres16050155

**Published:** 2026-10-09

**Authors:** Cristina Nazaré, Carla Matos Silva, Cláudia Reis, Margarida Serrano

**Affiliations:** Polytechnic University of Coimbra, Rua da Misericórdia, Lagar dos Cortiços, S. Martinho do Bispo, 3045-093 Coimbra, Portugal; cristinanazare@estesc.ipc.pt (C.N.);

**Keywords:** temporal auditory processing, mobile application, digital screening, Frequency Pattern Test, Duration Pattern Test, concurrent validity

## Abstract

**Highlights:**

**What is the main finding?**

- Abbreviated app-based DPT and FPT scores were positively associated with corresponding abbreviated 10-item BAPA-PE comparison measures in adults with normal peripheral hearing, with no statistically significant differences in paired scores between assessment formats.

**What is the implication of the main finding?**

- The findings provide preliminary support for the concurrent validity of the app-based temporal-pattern measures. Further studies should establish test–retest reliability and evaluate performance in larger and clinically diverse populations before screening or diagnostic applications are considered.

**Abstract:**

**Background/Objectives:** Digital technologies are increasingly used in audiological assessment; however, evidence supporting clinically evaluated mobile tools for temporal auditory processing screening based on Frequency Pattern Test (FPT) and Duration Pattern Test (DPT) paradigms remains limited. This study examined preliminary concurrent validity of digital FPT and DPT implementations in the Evollu Hear A4A mobile application against corresponding measures from the Bateria de Avaliação do Processamento Auditivo para o Português Europeu (BAPA-PE)—Battery for Auditory Processing Assessment in European Portuguese. **Methods:** Twenty-seven adults with normal hearing (20–58 years) completed app-based and BAPA-PE versions of the DPT and FPT. Ten four-stimulus sequences were administered for each test and format, yielding scores from 0 to 10. Paired scores were compared using Wilcoxon signed-rank tests, and concurrent validity was examined using Spearman’s rank-order correlations. **Results:** Median DPT scores were 8 for both the app-based (IQR = 3) and BAPA-PE (IQR = 2) measures, while median FPT scores were 9 (IQR = 2) for both formats. No statistically significant differences were observed between app-based and BAPA-PE scores for the DPT (Z = −0.650, *p* = 0.516) or FPT (Z = −0.408, *p* = 0.683). Significant positive correlations were found between formats for both the DPT (ρ = 0.665, *p* < 0.001) and FPT (ρ = 0.614, *p* < 0.001). **Conclusions:** The app-based DPT and FPT showed significant positive associations with their corresponding BAPA-PE reference measures, providing preliminary evidence of concurrent validity in adults with normal hearing. Further studies involving broader performance ranges and clinical populations should evaluate reliability, screening cut-offs, sensitivity, and specificity.

## 1. Introduction

Central auditory processing (CAP) and auditory processing disorders (APD) have been defined and operationalized through professional guidelines and consensus statements published by the American Speech-Language-Hearing Association (ASHA) [1,2], the American Academy of Audiology [3], Jerger and Musiek [4], and Iliadou et al. [5].

Central auditory processing refers to the efficiency and effectiveness with which the central nervous system processes, interprets, and organizes auditory information. Among the various auditory abilities involved in CAP, temporal processing plays a fundamental role in the perception and interpretation of time-related acoustic information. It enables the processing of rapid changes in sound, temporal patterns, temporal resolution, and sequences of auditory events that are essential for speech perception, music appreciation, and auditory scene analysis [1,2,6,7].

Temporal processing deficits have been associated with a range of developmental and neurological conditions and may contribute to difficulties in speech understanding, language development, and higher-order cognitive functions [8,9].

Consequently, the assessment of temporal auditory abilities constitutes an important component of central auditory processing evaluation. Within temporal processing, temporal ordering and sequencing refer to the ability to perceive and correctly identify the order of auditory events over time. The Frequency Pattern Test (FPT) and Duration Pattern Test (DPT) are widely established clinical measures of temporal sequencing and auditory pattern recognition [10,11,12,13].

The FPT evaluates the perception and sequencing of pitch variations, whereas the DPT assesses the perception and ordering of duration cues. Both tests are extensively used in clinical practice and have demonstrated sensitivity to central auditory processing dysfunction [10,11,12,13]. However, their administration generally requires specialized equipment, controlled testing conditions, and trained personnel, which may limit accessibility and restrict the implementation of large-scale screening initiatives, particularly in underserved populations [14].

In recent years, advances in telemedicine and digital health have facilitated the remote delivery of healthcare services through mobile and wireless technologies. Mobile health (mHealth), defined as the use of mobile devices to support medical and public health practice, has emerged as an important component of digital healthcare, enabling the delivery of health services beyond traditional clinical settings [15,16]. In audiology, telehealth applications have been successfully implemented for hearing aid fitting, programming, counselling, and follow-up care. However, the remote assessment of auditory function, particularly for diagnostic purposes, remains less established and requires further validation before widespread clinical implementation [17].

Advances in mHealth and digital audiology have created new opportunities for the development of accessible, scalable, and cost-effective auditory assessment tools. The widespread availability of smartphones has facilitated the implementation of digital solutions for hearing screening, assessment, training, and rehabilitation. Nevertheless, the clinical adoption of mobile-based auditory assessment tools depends on evidence demonstrating that they provide valid and reliable measurements comparable to those obtained with established clinical assessment methods across different populations and testing environments [18,19,20,21].

Despite the growing number of digital audiology applications, evidence supporting validated and clinically evaluated mobile tools for temporal auditory processing screening based on the Frequency Pattern Test and Duration Pattern Test paradigms remains limited. Addressing this gap may facilitate access to auditory screening services, support the early identification of individuals at risk for auditory processing difficulties, and improve referral pathways for comprehensive audiological evaluation [20,21]. More broadly, the development of such tools aligns with ongoing efforts to expand auditory processing assessment resources for European Portuguese speakers, including the adaptation of test materials and instructions where linguistic considerations are relevant [22,23].

To address this need, the Audiology for All (A4A) Project developed the Evollu Hear A4A App, a mobile platform that incorporates hearing-related assessments, including measures of listening effort [24] and central auditory processing. The application incorporates digital implementations of the Frequency Pattern Test (FPT) and Duration Pattern Test (DPT) designed for screening purposes, with automated scoring and result reporting. Its computerized administration uses a restricted-response paradigm with adjustable levels of difficulty, minimizing attentional and memory demands while requiring only simple decision-making and motor responses. This design is intended to reduce the influence of non-auditory cognitive factors and facilitate the differentiation of auditory processing deficits from language, attention, and other cognitive difficulties [25,26].

For validation purposes, the corresponding FPT and DPT measures from the BAPA-PE battery [Bateria de Avaliação do Processamento Auditivo para o Português Europeu (BAPA-PE)—Battery for Auditory Processing Assessment in European Portuguese] were selected as reference measures. The BAPA-PE is a standardized battery widely used for assessing auditory processing abilities in European Portuguese speakers [23,27].

Therefore, the present study aimed to examine preliminary evidence of concurrent validity for the digital FPT and DPT implemented in the Evollu Hear A4A mobile application by comparing performance outcomes with those obtained using the corresponding BAPA-PE reference measures. It was hypothesized that performance on the digital tests would be significantly associated with performance on the corresponding reference measures.

## 2. Materials and Methods

**Ethical considerations**

Prior to data collection, ethical approval was obtained from the Ethics Committee of the Polytechnic Institute of Coimbra (approval number: 94_CEIPC/2021). Written and verbal informed consent was obtained from all participants before enrolment in the study. All procedures were conducted in accordance with the principles of the Declaration of Helsinki.

**Study design**

This was a cross-sectional validation study designed to examine the concurrent validity of the app-based Duration Pattern Test (DPT) and Frequency Pattern Test (FPT) using the corresponding BAPA-PE tests as reference measures.

**Participants**

Participants were recruited by convenience sampling among students and staff of the institution. No a priori sample-size calculation was performed. The sample size was determined by participant availability during the recruitment period, consistent with the preliminary nature of the study. A total of 27 adults participated in the study, with a mean age of 41.74 years (range: 20–58 years). Of these, 7 (25.9%) were male and 20 (74.1%) were female. Prior to participation, all individuals underwent otoscopic examination using a HEINE 2000 otoscope, tympanometry by an Inventis Clarinet clinical middle ear analyzer with a 226 Hz probe tone, and pure-tone audiometry with a GSI Pello audiometer with DD450 headphones. Air-conduction thresholds were determined using a 10 dB down, 5 dB up procedure at frequencies from 125 to 8000 Hz, including 6000 Hz.

Inclusion criteria were bilateral type A or C1 tympanograms, with type C1 defined as a middle-ear peak pressure between −100 and −200 daPa; air-conduction hearing thresholds of 15 dB HL or better at all frequencies from 125 to 8000 Hz, including 6000 Hz; being a native speaker of European Portuguese, to ensure consistency in the verbal response requirements of the BAPA-PE; no history of formal musical training; and the ability to understand the test instructions. None of the participants presented a type C1 tympanogram; all participants had bilateral type A tympanograms. Bone-conduction testing was therefore not required.

**Procedures**

The Evollu Hear A4A App was developed for iOS to enable the automated administration of listening effort and auditory processing screening tests through a touchscreen interface. The app-based Duration Pattern Test (DPT) and Frequency Pattern Test (FPT) were developed based on the temporal pattern perception literature and the auditory temporal ordering paradigms originally described by Musiek and colleagues [11,12,19].

Four-stimulus versions of the DPT and FPT were implemented and evaluated in the present study.

The app-based DPT consisted of 10 sequences of four 1000 Hz tone bursts differing only in duration. Stimuli consisted of short (250 ms) and long (500 ms) tones, with an interstimulus interval (ISI) of 300 ms and a 10 ms rise/fall time. Participants were required to identify the order of the tones within each sequence (e.g., short–long–short–long).

The app-based FPT consisted of 10 sequences of four 250 ms tone bursts differing in frequency. Stimuli were presented at 880 Hz (low) and 1430 Hz (high), with an ISI of 300 ms and a 10 ms rise/fall time. These frequencies were selected based on equal-loudness contour principles [28] to minimize perceived loudness differences between the low- and high-frequency stimuli. Participants were required to identify the order of the frequencies within each sequence (e.g., low–high–low–high).

To examine concurrent validity, participants also completed the corresponding DPT and FPT from the BAPA-PE battery, which served as reference measures. The tests were administered following the procedures described in the BAPA-PE manual [27], except for the number of items presented. In the BAPA-PE, stimulus sequences are randomly selected from the set of possible sequences and presented by the BAPA-PE system. For the present study, administration was discontinued after the first 10 sequences selected by the system for each test, thereby matching the number of items and scoring range of the app-based tests. This adaptation allowed direct comparison between the app-based and reference measures using comparable test lengths and scoring ranges.

The purpose of this comparison was to assess concurrent validity rather than to establish normative performance or reproduce the full standardized BAPA-PE protocol. Both assessment formats included the same range of possible four-stimulus patterns, although the specific sequences presented were not necessarily identical. The reduced administration of the BAPA-PE tests should therefore be regarded as a methodological adaptation for the purposes of the present study rather than as a modification of the standardized protocol for clinical use.

For both assessment formats, performance was quantified as the number of correctly identified sequences, yielding a maximum score of 10 for each test.

Participants completed the app-based and BAPA-PE tests during the same session. The order of administration was counterbalanced across participants to minimize potential order, practice, and fatigue effects. Before formal testing, participants completed the practice items provided in each assessment format. These familiarization trials followed the procedures specified for each test and were not included in the final scores. Their purpose was to ensure that participants understood the task before data collection.

Responses were not time-limited in either assessment format, and trials did not expire or advance automatically in the absence of a response. In the app-based tests, participants manually advanced to the next trial after providing their response, whereas in the BAPA-PE, progression to the next trial was controlled by the examiner. Stimulus sequences were presented only once and could not be repeated in either assessment format.

All assessments were conducted in a sound-treated booth at the Audiology Laboratory of the Coimbra Health School, Polytechnic University of Coimbra, Portugal.

**Equipment and test administration**

BAPA-PE stimuli were presented binaurally at 60 dB HL using a GSI Pello audiometer (Grason-Stadler, Eden Prairie, MN, USA) with DD450 headphones. The audiometer was calibrated in accordance with ISO 389-8:2004 [29] before data collection.

To approximate presentation levels across assessment formats, the acoustic output of the audiometer-based BAPA-PE system at 60 dB HL was measured using a Brüel & Kjær Type 2238 Mediator sound level meter, without an acoustic coupler. The output of the app-based system was then adjusted to obtain a comparable overall sound-pressure level under the same measurement conditions. This procedure represented a global level adjustment rather than a formal headphone calibration, and the individual stimulus frequencies were not separately verified. The app-based tests were administered using an iPhone 12 running iOS 16.2 and Corsair HS70 Bluetooth headphones. The resulting device volume setting was kept constant across all participants.

Responses to the app-based tests were entered directly on the touchscreen by selecting the stimulus pattern corresponding to the sequence presented. In the BAPA-PE tests, responses were provided orally and scored by the examiner.

**Outcome measures**

For each test, one point was awarded for each correctly identified sequence, yielding a total score ranging from 0 to 10. The number of correct responses obtained for the app-based and BAPA-PE versions of the DPT and FPT was recorded for each participant and used in the concurrent validity analyses.

**Statistical analysis**

Statistical analyses were performed using IBM SPSS Statistics (Version 30; IBM Corp., Armonk, NY, USA). Descriptive statistics, including medians, interquartile ranges (IQRs), and ranges (minimum–maximum), were calculated for the app-based and BAPA-PE versions of the Duration Pattern Test (DPT) and Frequency Pattern Test (FPT).

Given the small sample size and the bounded and discrete nature of the outcome measures, with scores ranging from 0 to 10, non-parametric statistical procedures were used. Wilcoxon signed-rank tests were performed to compare paired scores obtained with the app-based and corresponding BAPA-PE versions of each test.

To examine concurrent validity, Spearman’s rank-order correlation coefficients (ρ) were calculated to assess the association between scores obtained with the app-based DPT and FPT and those obtained with the corresponding BAPA-PE reference measures. For Spearman’s correlation coefficients, 95% confidence intervals were estimated using bias-corrected and accelerated (BCa) bootstrap resampling with 20,000 paired resamples.

All statistical tests were two-tailed, and statistical significance was set at *p* < 0.05.

## 3. Results

The results are presented in terms of descriptive statistics, comparisons between the app-based and BAPA-PE assessment formats, and associations between the app-based and reference measures.

### 3.1. Descriptive Statistics

Descriptive statistics for the app-based and BAPA-PE versions of the Duration Pattern Test (DPT) and Frequency Pattern Test (FPT) are presented in Table 1 and Figure 1. Results are summarized using medians, interquartile ranges (IQRs), and minimum–maximum values.

### 3.2. Comparison Between Assessment Formats

Wilcoxon signed-rank tests were used to compare paired scores obtained with the app-based and BAPA-PE versions of each test. No statistically significant difference was observed between the app-based and BAPA-PE DPT scores (Z = −0.650, *p* = 0.516). Similarly, no statistically significant difference was observed between the app-based and BAPA-PE FPT scores (Z = −0.408, *p* = 0.683).

### 3.3. Concurrent Validity

Concurrent validity was examined using Spearman’s rank-order correlations between scores obtained with the app-based tests and their corresponding BAPA-PE reference measures.

A significant positive correlation was observed between app-based and BAPA-PE DPT scores (ρ = 0.665, 95% CI [0.393, 0.833], *p* < 0.001). Similarly, app-based and BAPA-PE FPT scores were significantly positively correlated (ρ = 0.614, 95% CI [0.250, 0.837], *p* < 0.001) (Figure 2).

## 4. Discussion

The present study examined preliminary evidence of concurrent validity for digital implementations of the Duration Pattern Test (DPT) and Frequency Pattern Test (FPT) integrated into the Evollu Hear A4A mobile application. Significant positive correlations were observed between the app-based tests and their corresponding BAPA-PE reference measures for both the DPT and FPT. In addition, no statistically significant differences were observed between scores obtained with the two assessment formats. Together, these findings provide preliminary support for the concurrent validity of the digital measures in adults with normal hearing.

The positive correlations observed for both temporal patterning tasks indicate that relative performance was associated across assessment formats. Within the range observed in the present sample, lower and higher scores on the BAPA-PE measures tended to correspond to lower and higher scores, respectively, on the app-based tests. This finding is consistent with previous research demonstrating the feasibility of computerized approaches to auditory assessment. Lelo de Larrea-Mancera et al. [18], using the Portable Automated Rapid Testing (PART) platform, demonstrated the potential of automated procedures for assessing auditory abilities, while de Carvalho et al. [19] investigated computerized procedures for auditory processing screening. Smartphone-based approaches have also been explored for hearing assessment and screening in different populations and settings [30,31]. The present findings extend this body of work by providing preliminary concurrent validity data for digitally administered temporal patterning tasks.

No statistically significant differences were observed between the app-based and BAPA-PE scores for either the DPT or FPT. Although this finding does not establish equivalence between the assessment formats, it provides complementary information to the correlation analyses, indicating that no systematic differences in paired scores were detected in the present sample. This is relevant given the different response modalities used: responses to the BAPA-PE measures were provided orally, whereas responses to the app-based tests were entered through touchscreen selection. Temporal patterning tasks involve auditory discrimination and temporal ordering, together with attentional and cognitive demands [6,12], and differences in response requirements should therefore be considered when comparing administration formats.

The use of a mobile platform may offer practical advantages through automated administration and scoring and is consistent with broader developments in digital audiology and technology-assisted hearing healthcare [32]. However, the present study examined concurrent validity rather than screening accuracy. Although the Evollu Hear A4A application is intended to support screening and referral, establishing its performance for this purpose will require investigation in populations representing a broader range of auditory processing abilities. Such studies will be necessary to determine appropriate screening cut-off values and evaluate sensitivity and specificity.

The findings should be interpreted considering the study sample and design. No a priori sample-size calculation was performed, and the relatively small sample consisted of adults with normal hearing who generally achieved high scores, resulting in a restricted range of observed performance. In addition, 10 items from each BAPA-PE test were administered to match the length and scoring range of the app-based tests rather than using the complete standardized protocol. The different response modalities used by the two formats should also be considered. Stimulus durations, interstimulus intervals, and rise/fall times were also not acoustically verified at the Bluetooth headphone output; therefore, potential effects of Bluetooth transmission on the temporal characteristics of the stimuli cannot be excluded.

**Future directions**

Future studies should therefore include larger and more heterogeneous samples, particularly individuals with suspected or confirmed auditory processing difficulties, to determine whether the associations observed here are maintained across a broader performance continuum. Test–retest reliability should also be investigated as an additional psychometric property of the digital measures [33], followed by studies examining screening cut-offs, sensitivity, and specificity.

## 5. Conclusions

The app-based DPT and FPT showed significant positive associations with their corresponding BAPA-PE reference measures, providing preliminary evidence of concurrent validity in adults with normal hearing. These findings support further investigation of digitally administered temporal patterning tests. Studies involving larger and more diverse populations, together with evaluation of reliability and screening accuracy, are needed to establish their potential role in auditory processing screening.

## Figures and Tables

**Figure 1:**
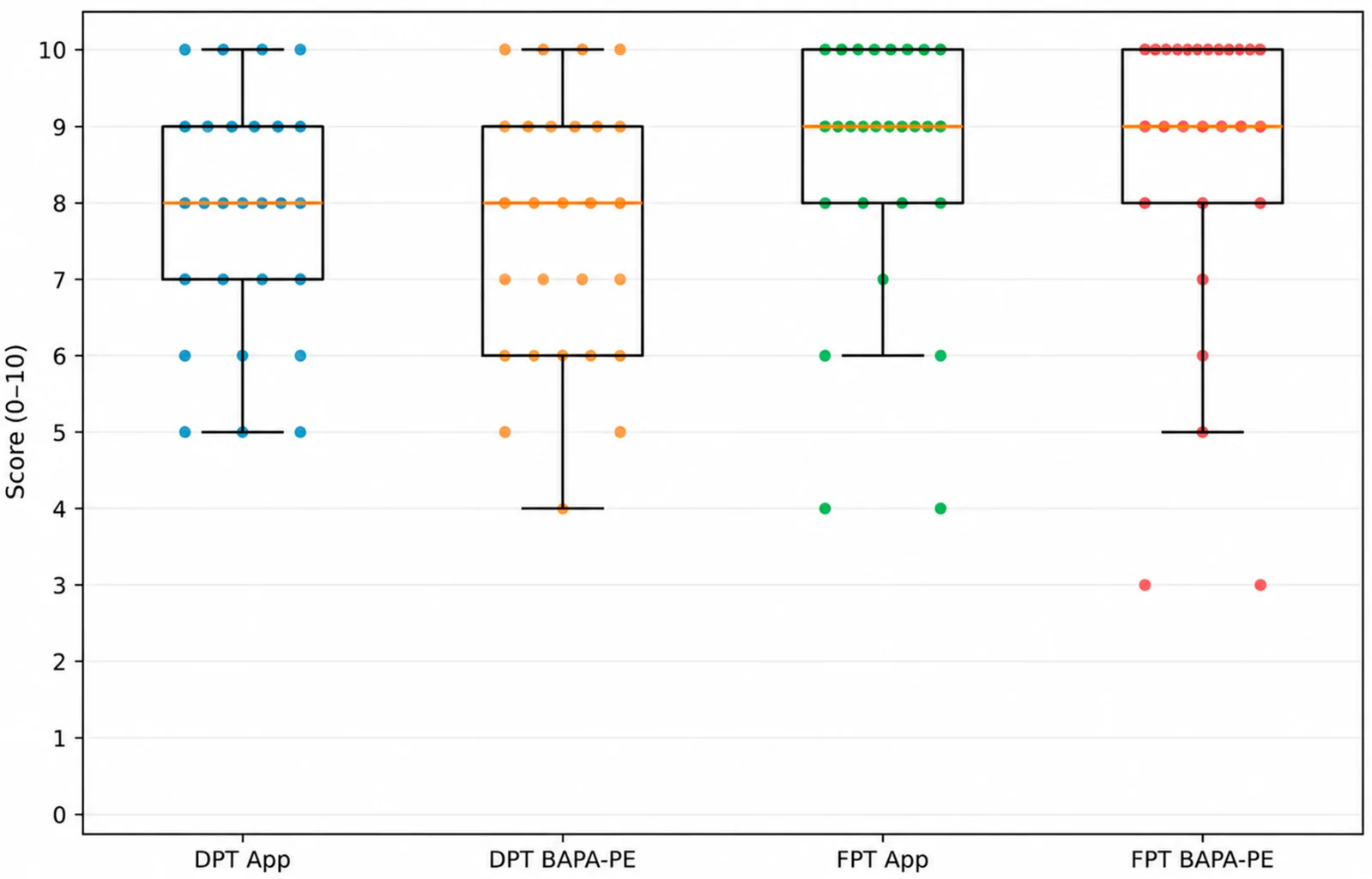
Distribution of DPT and FPT scores obtained with the app-based and BAPA-PE assessment formats. Box-and-whisker plots are shown with individual participant scores overlaid. Scores range from 0 to 10, with higher scores indicating better performance.

**Figure 2:**
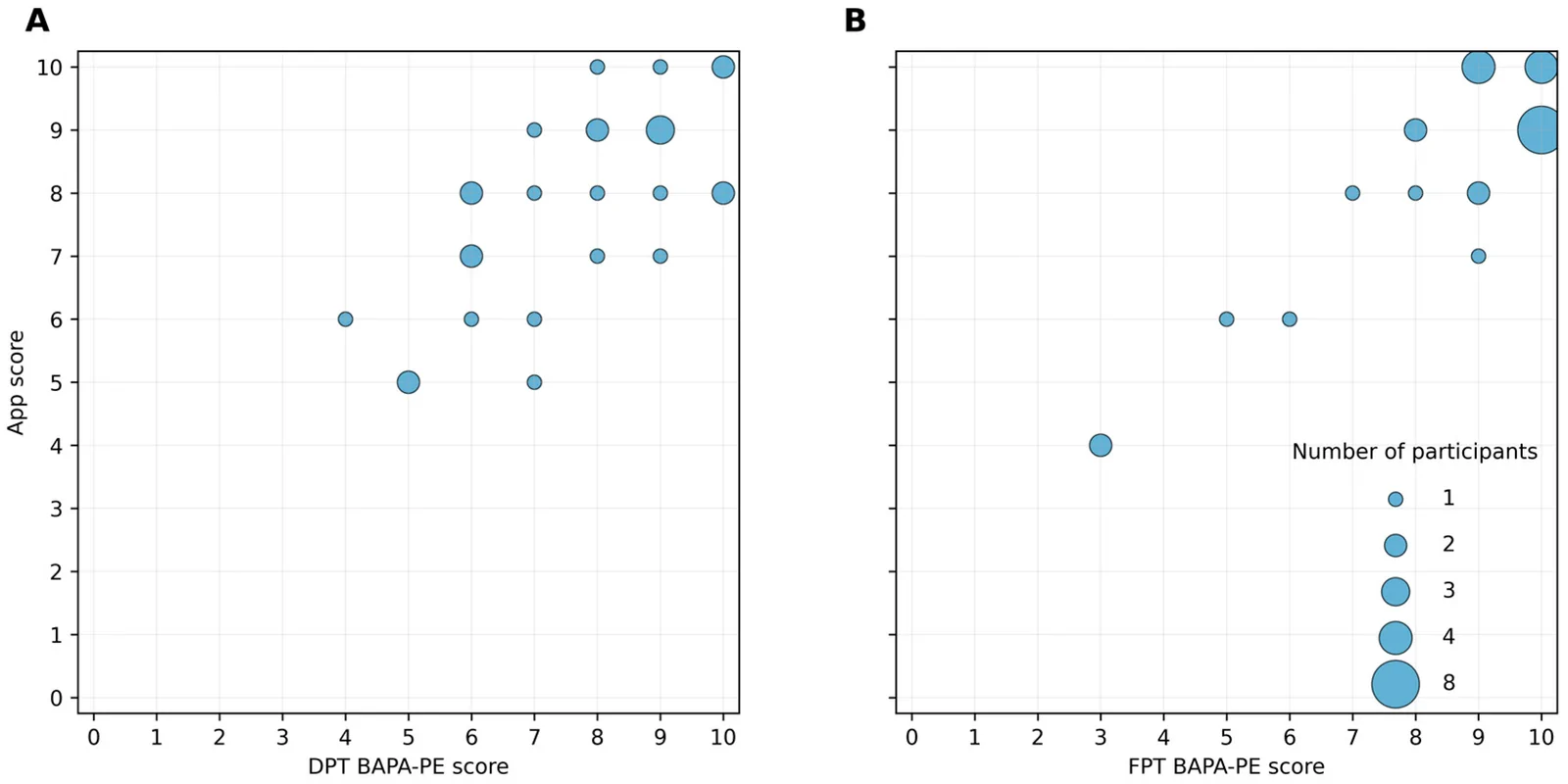
Association between app-based and BAPA-PE scores for (**A**) the Duration Pattern Test (DPT) and (**B**) the Frequency Pattern Test (FPT). Bubble size represents the number of participants with each observed score pair.

**Table 1:** Descriptive statistics for app-based and BAPA-PE DPT and FPT scores.

Test	Assessment Format	Median	IQR	Minimum	Maximum
DPT	App-based	8.00	3.00	4	10
DPT	BAPA-PE	8.00	2.00	5	10
FPT	App-based	9.00	2.00	3	10
FPT	BAPA-PE	9.00	2.00	4	10

## Data Availability

The data supporting the findings of this study are not publicly available but are available from the corresponding author upon reasonable request.
